# Technical aspects and early results of uniportal video-assisted thoracoscopic complex segmentectomy: a 30 case-series study

**DOI:** 10.1186/s13019-022-01808-8

**Published:** 2022-04-02

**Authors:** Xiang-Long Kong, Jun Lu, Peng-Ju Li, Bo-Xiong Ni, Kai-Bin Zhu, Hai Xu, Shi-Dong Xu

**Affiliations:** Department of Thoracic Surgery, Harbin Medical University Cancer Hospital, No. 150, Hapin Road, Harbin, 150081 China

**Keywords:** Uniportal video-assisted thoracic surgery, Complex segmentectomy, Subsegmentectomy, Three-dimensional computed tomography

## Abstract

**Background:**

With the advantages of better cosmetic incision and faster recovery, uniportal video-assisted thoracoscopic surgery (UP-VATS) has developed rapidly worldwide in recent decades, and indications for UP-VATS have been further expanded to those for conventional VATS. Complex segmentectomy that makes several or intricate intersegmental planes, with more complex procedures, continues to be difficult in minimally invasive techniques. However, there are few reports on UP-VATS complex segmentectomy. In this report, we describe the perioperative clinical data and operative techniques and present our early results of UP-VATS complex segmentectomy in our hospital.

**Methods:**

The records of a total of 30 patients who underwent UP-VATS complex segmentectomy by a single surgeon between January 2021 and June 2021 were retrospectively reviewed. We defined cases as complex segmentectomy if they required resection of segments 9 and 10, combined segmentectomy, segmentectomy + subsegmentectomy, subsegmentectomy, or combined subsegmentectomy.

**Results:**

The mean age was 52.8 ± 9.9 years old; the mean nodule size was 0.84 ± 0.36 cm; the mean margin width was 2.307 ± 0.309 cm; the median operative time was 229.0 ± 58.06 min; the mean operative hemorrhage was 56.60 ± 17.95 mL; 5.58 ± 1.74 lymph nodes dissected had not metastasized; the mean duration of postoperative chest tube drainage was 4.7 ± 1.4 days; and the mean postoperative hospital stay was 6.5 ± 3.0 days. Although 1 patient experienced a prolonged air leak, the other 29 recovered uneventfully. Another patient failed to reach the 2-cm safe margins and subsequently underwent completion lobectomy.

**Conclusions:**

UP-VATS complex segmentectomy is a safe and effective procedure in the treatment of lung cancers, sparing more pulmonary parenchyma and ensuring safe margins, with the disadvantage being the lengthy operative times during early skill acquisition.

## Introduction

For benign lesions and ground glass opacity (GGO)-dominant early-stage lung cancer, the current trend is precise resection with sufficient surgical margins and more preservation of pulmonary function [1–4]. Anatomic segmentectomy in treating early-stage non-small-cell lung cancer (NSCLC) is chosen more frequently because of the same oncological outcomes as lobectomy and the sufficient surgical margin [5, 6].

A study based on high-resolution computed tomography reported that more than 33% of c-T1aN0M0 pulmonary nodules involved multiple segments [7]. To ensure an adequate surgical margin, complex segmentectomy has been applied in certain clinical circumstances. Complex segmentectomy creates several or intricate intersegmental planes with a more complex procedure [4, 7–9]. Currently, few articles have addressed complex segmentectomy, a procedure that may be particularly challenging in the setting of UP-VATS, as the dexterity and fineness of instrument operation under UP-VATS are limited [10–12].

Our main goal is to evaluate the technical feasibility of UP-VATS complex segmentectomy and to analyze the early clinical results when using this procedure.

## Materials and methods

### Patient population

The Ethics Committee of Harbin Medical University Cancer Hospital approved this study because of its retrospective design. From January 2021 to June 2021, 30 UP-VATS complex segmentectomies were performed for benign lesions and ground glass opacity (GGO)-dominant early-stage lung cancer by a team at the Thoracic Surgery Department of Harbin Medical University Cancer Hospital.

Complex segmentectomy was defined as segmental resection of segments 9 and 10, combined segmentectomy, segmentectomy + subsegmentectomy, subsegmentectomy, or combined subsegmentectomy.

All 30 patients fulfilled the following criteria: (1) The maximum diameter of the pulmonary nodule detected on CT reconstruction lung window imaging was required to be ≤ 2 cm with a ≥ 50% ground glass opacity appearance on CT. (2) The lesion was far away from the visceral pleura, so it was difficult to ensure a safe margin with wedge resection. (3) Intraoperative frozen negative lymph nodes were found in lymph nodes 10, 11, 12 and 13, and the estimated margin width was ≥ 2 cm. (4) Compromised surgery or benign tumors may be involved.

The clinical data of patients undergoing UP-VATS complex segmentectomy were retrospectively reviewed for age, sex, resected region, operative time, operative blood loss, final pathologic diagnosis, duration of chest tube placement, hospitalization, and intraoperative and postoperative complications. Each patient underwent enhanced thin-section (1-mm) thoracic CT. The reconstruction software “Mimics” was used to reconstruct images of the pulmonary bronchi and blood vessels.

### Operative techniques

**Fig. 1:**
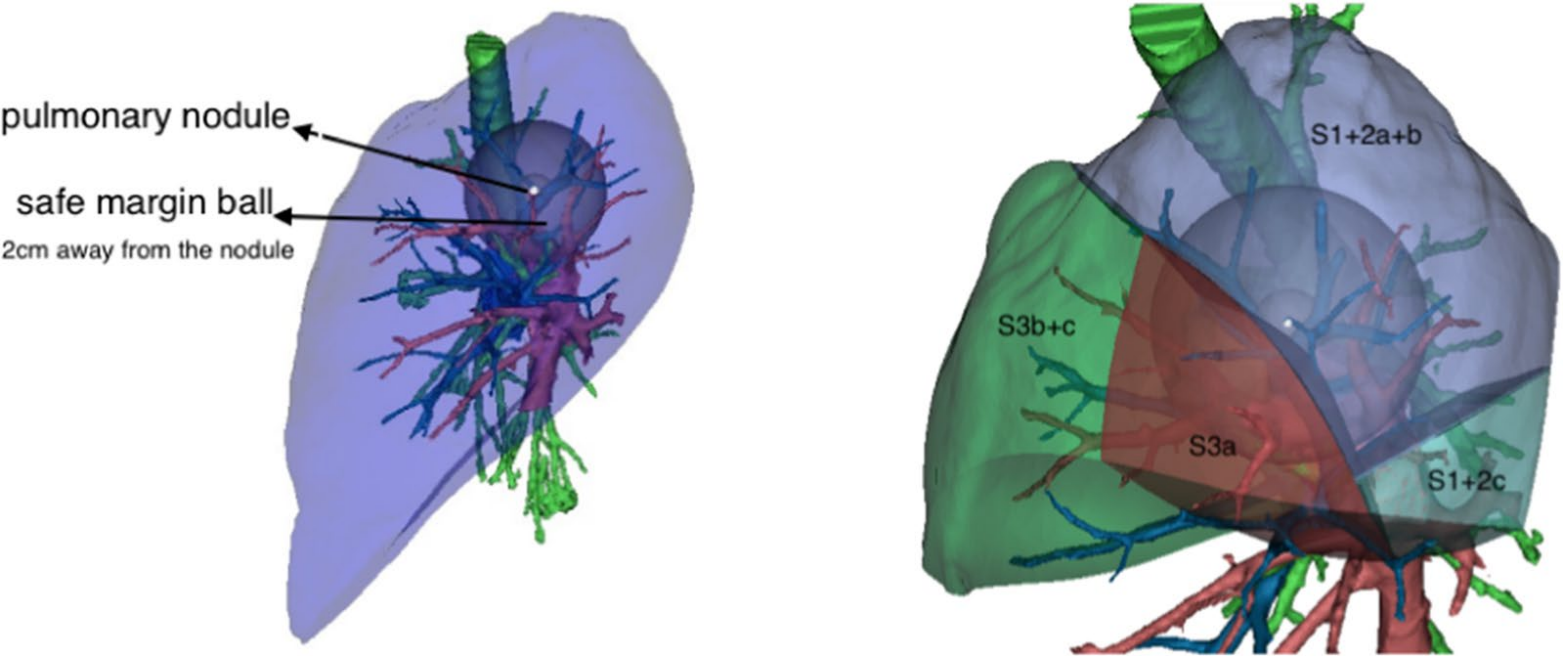
Complex segmentectomy based on nodule-centered surgical planning with parenchymal resection margins ≥ 2 cm and subsegment as a surgical unit with the guidance of three-dimensional (3D) navigation

**Fig. 2:**
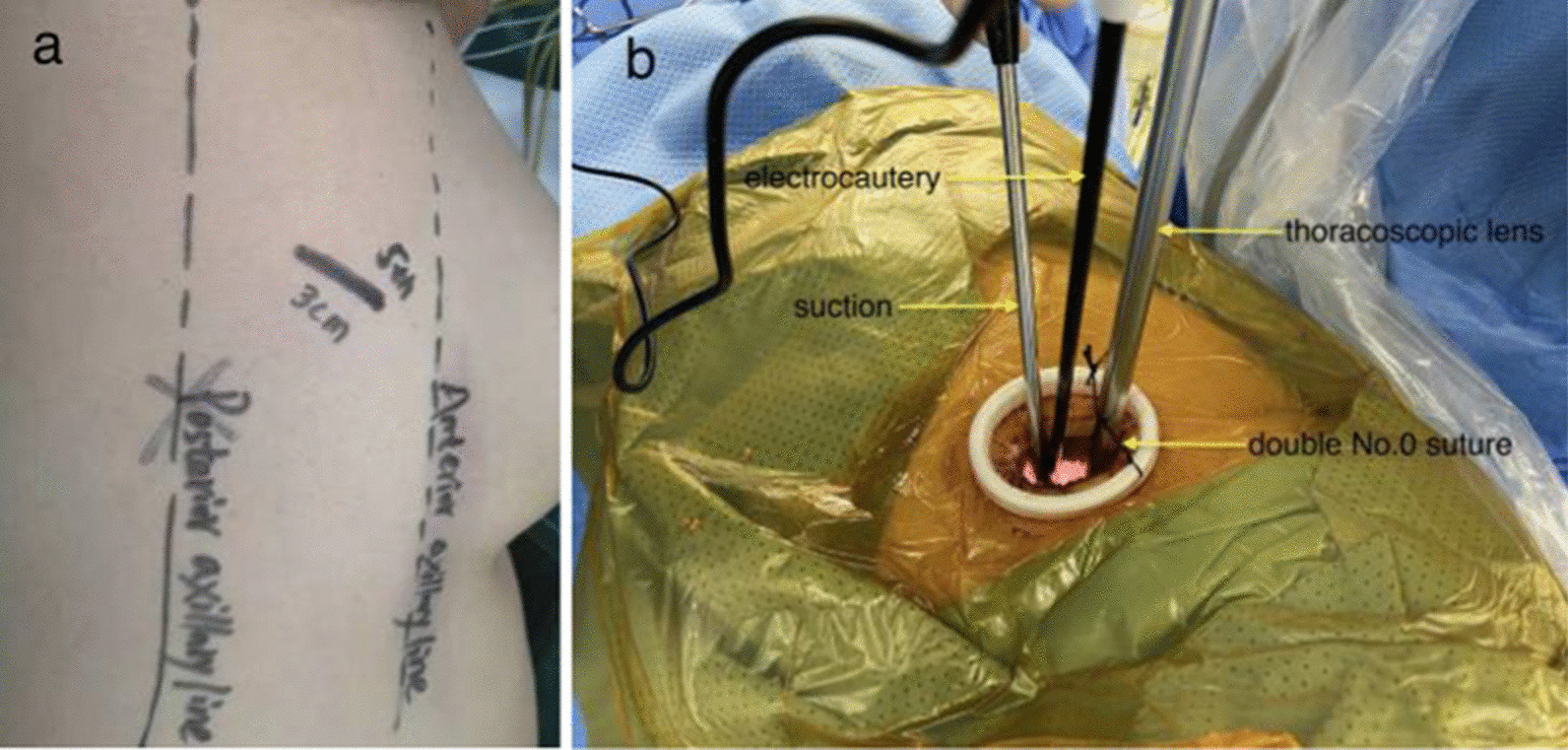
**a** Body surface location of the surgical incision. **b** The assistant stood on the opposite side of the operator and held the thoracoscopic lens, which was limited by the double No. 0 suture

Complex segmentectomies were planned and performed under the guidance of 3D navigation. Tumor locations, segmental structures and all bronchi, arteries, and veins involved were confirmed. To achieve an adequate surgical margin, we designed the complex segmentectomy based on nodule-centered surgical planning with parenchymal resection margins ≥ 2 cm and subsegment as a surgical unit (Fig. 1). An incision approximately 3.0 cm in length was made at the 5th intercostal space between the anterior-axillary line and posterior-axillary line and protected with a wound retractor (Fig. 2a, b). The specific location of the incision depends on the anatomic location of the hilum of the target segment. It is best to strive for a 45 degree angle between the operation angle of the surgical instrument and the hilar of the segment.

**Fig. 3:**
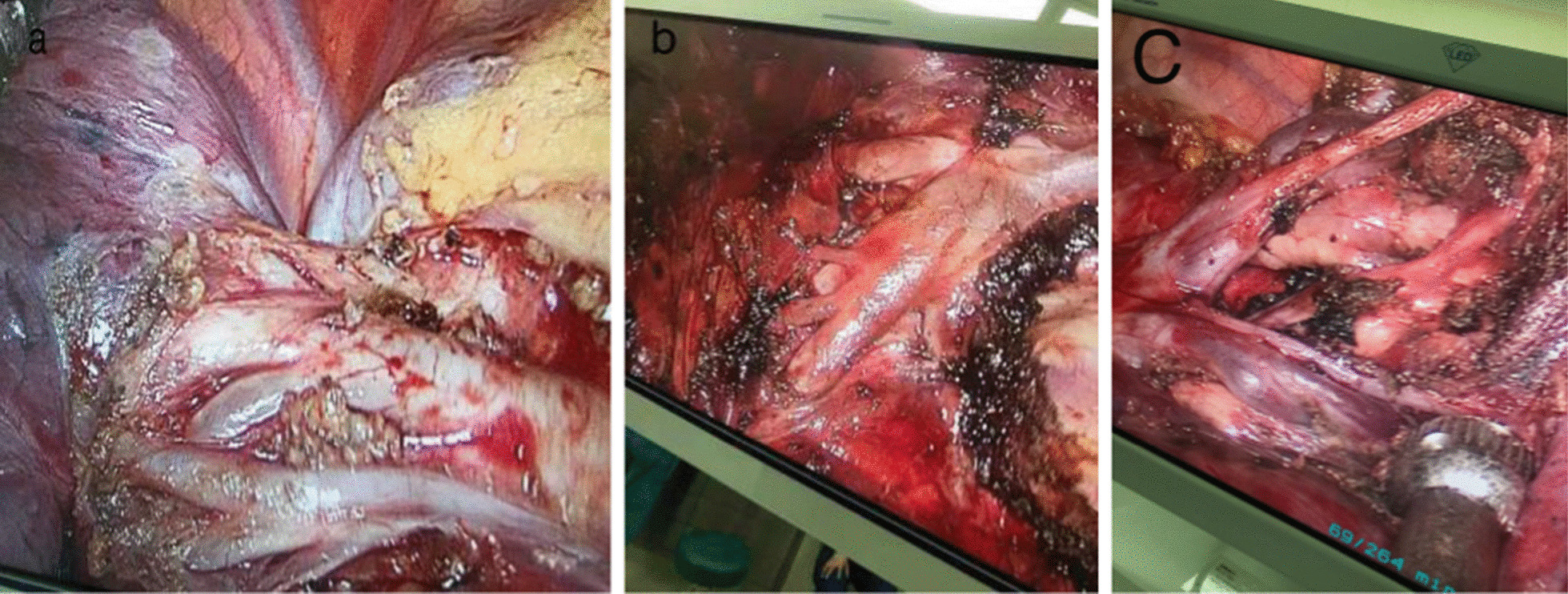
**a** Arteriovenous dissection and lymph node sampling of the right upper lobe. **b** Arterial dissection and lymph node sampling of the right lower lobe. **c** Bronchovascular dissection and lymph node sampling of the left upper lobe

**Fig. 4:**
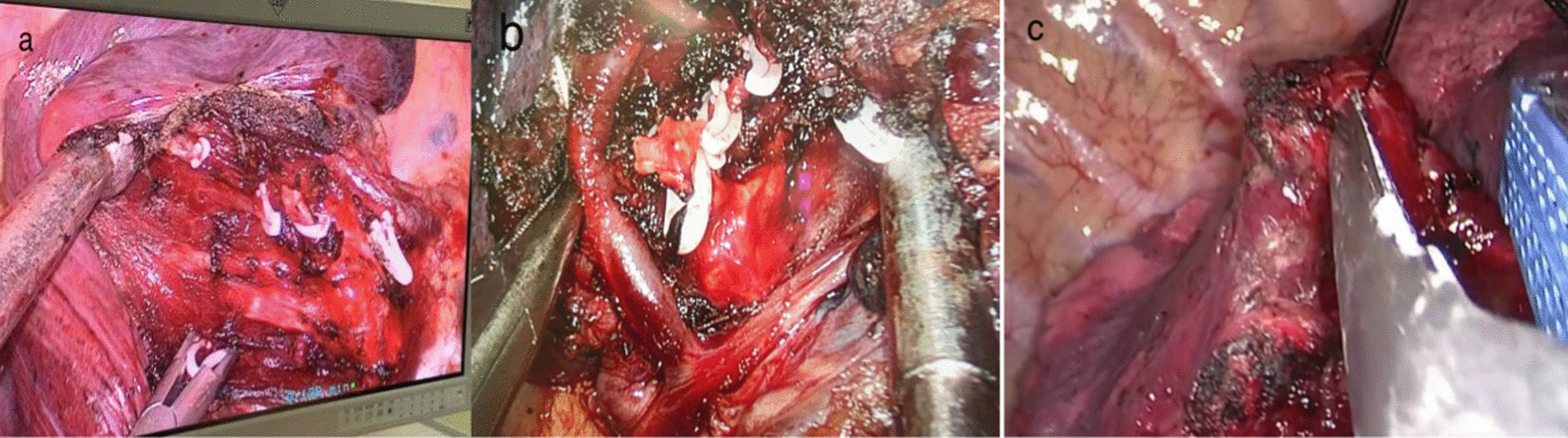
The target bronchus was transected using a stapler or Hem-o-lok

**Fig. 5:**
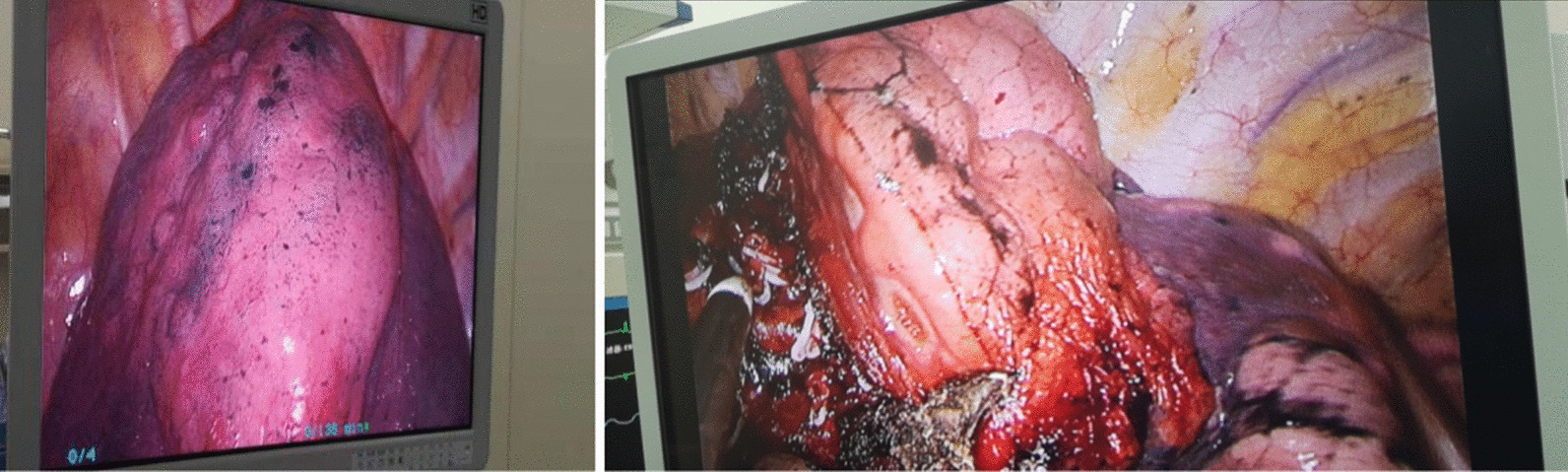
A modified “inflation-deflation” technique was used to identify the intersegmental border

**Fig. 6:**
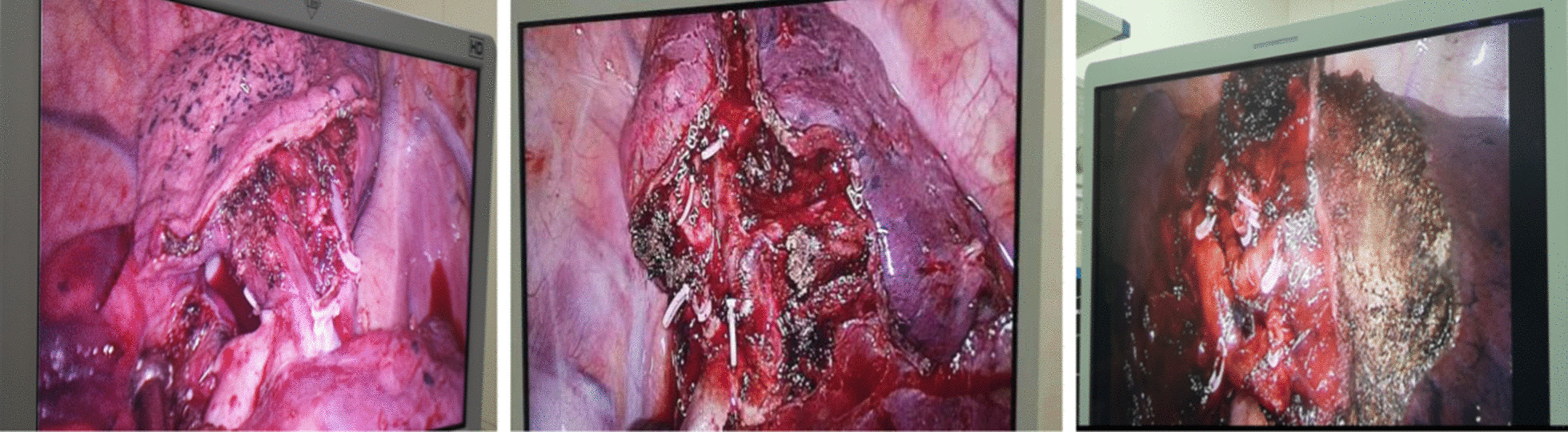
Combined application of energy devices and staplers for the management of the intersegmental plane

**Fig. 7:**
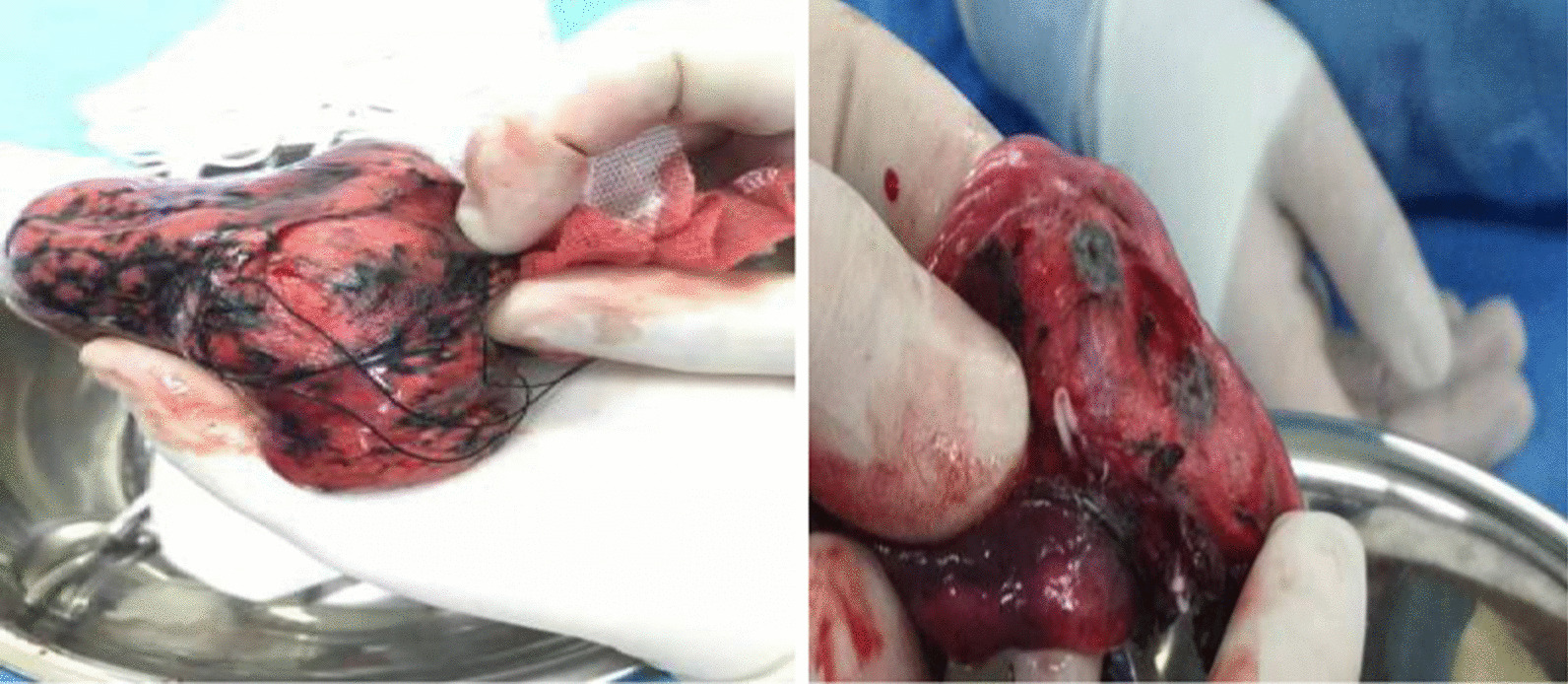
Safe margin width ≥ 2 cm

An electric hook with two curvatures and an ultrasonic scalpel were used for bronchovascular dissection and No. 10, 11, 12 and 13 lymph node sampling (Fig. 3a, b). The target bronchus was transected using a stapler or Hem-o-lok (Fig. 4). The target vessels can be stapled, ligated, or clipped according to the specific conditions. A modified “inflation-deflation” technique was used to identify the intersegmental border thereafter (Fig. 5), enabling dissection of intersegmental planes using electrocautery, ultrasonic scalpel, or staple along the intersegmental veins (Fig. 6). An air leak test was performed, and fibrin glue was applied to reduce postoperative air leakage. The margin width (from the resection margin to the nodule detected in the removed specimen) was measured by the surgeon intraoperatively (Fig. 7).

## Results

The clinical characteristics of the 30 patients (9 men and 21 women, mean age 52.8 ± 9.9, range: 32–71 years) and the details of the nodule locations and surgical procedures in this study are presented in Table 1. The mean nodule size was 0.84 ± 0.36 cm. The lesions were located in the right upper lobe (13, 43.3%), the left upper lobe (11, 36.6%), and the lower lobe (6, 20.0%).

**Table 1:** Clinical characteristics of the patients, nodule locations and surgical procedures

Factor	Complex segmentectomy
Age	
Mean (range)	52.8 ± 9.9 (32–71 years)
Sex	
Male	9
Female	21
Mean nodule size (cm)	0.84 ± 0.36
Nodule location	
RUL	13
S^1^ + S^2^a	3
S^2^ + S^3^a	1
S^3^ + S^1^b	3
S^1^ + S^2^ + S^3^a	1
S^1^b	1
S^3^b	1
S^2^b + S^3^a	2
S^1^ + S^2^ + S^3^ai + bi	1
LUL	11
S^1+2^	1
S^1+2^a + b	2
S^1+2^a + b + S^3^c	2
S^1+2^b	1
S^1+2^c + S^3^a + b	1
S^1+2^c + S^3^a	1
S^3^ + S^1+2^a	2
S^3^a + S^4^a	1
RLL	2
S^6^b + S^8^a + S^9^a	1
S^10^	1
LLL	4
S^6^ + S^8^a	1
S^8^a + S^9^a	1
S^7^ + S^8^	1
S^9^ + S^10^	1

RUL, right upper lobe; LUL, left upper lobe; RLL, right lower lobe; LLL, left lower lobe

The evaluation of intraoperative and postoperative factors is shown in Table 2. No patients required conversion to thoracotomy. The median operative time was 175 min (range, 75 to 294 min); the mean operative hemorrhage was 56.60 ± 17.95 mL; 5.58 ± 1.74 lymph nodes dissected had not metastasized; the mean duration of postoperative chest tube drainage was 4.7 ± 1.4 days; and the mean postoperative hospital stay was 6.5 ± 3.0 days. Aside from one instance of prolonged air leakage (> 7 days), all patients recovered uneventfully. Another patient failed to reach the 2-cm safe margins and subsequently underwent completion lobectomy. No deaths occurred. The tumor pathological diagnoses included invasive adenocarcinoma (10 cases), minimally invasive adenocarcinoma (9 cases), adenocarcinoma in situ (6 cases), atypical adenomatous hyperplasia (3 cases), and benign (2 cases).

**Table 2:** Evaluation of intraoperative and postoperative factors

Factor	Complex segmentectomy
Mean margin width (cm)	2.307 ± 0.309
Average operative duration (minutes)	229.0 ± 58.06
Operative hemorrhage (mL)	56.60 ± 17.95
Number of lymph nodes dissected	5.58 ± 1.74
Duration of postoperative chest tube drainage (days)	4.7 ± 1.4
Postoperative hospital stay (days)	6.5 ± 3.0
Pathological diagnoses	
Benign	2
AAH	3
AIS	6
MIA	9
IAC	10

AAH, atypical adenomatous hyperplasia; AIS, adenocarcinoma in situ; IAC, invasive adenocarcinoma; MIA, minimally invasive adenocarcinoma

## Discussion

Segmentectomy can be subdivided into simple and complex segmentectomy according to the condition of the intersegmental boundaries [8]. The ability to obtain a ≥ 2-cm safe surgical margin is the key factor that makes complex segmentectomy superior to simple segmentectomy [7, 11, 13]. With the use of the reconstruction software “Mimics”, we designed a complex segmentectomy based on nodule-centered surgical planning with parenchymal resection margins ≥ 2 cm and a subsegment as a surgical unit.

In our study, the margin width (2.307 ± 0.309 cm) met the surgical requirement, except for one patient who received completion lobectomy later. The advantage of complex segmentectomy is that it can achieve a sufficient margin for the great majority of nodules.

It is generally thought to be more difficult to divide complex segments when instruments are operated in a single direction or are limited by angle changes in UP-VATS [11, 14, 15]. However, with accumulated experience and familiarity with anatomy and operation techniques, UP-VATS complex segmentectomy can be performed safely [15–17]. The following tips on technical aspects are helpful if we encounter difficulties during UP-VATS complex segmentectomy.

1. Further dissection of the segmental hilar structures
  It is important to dissect the surrounding lung parenchyma and lymph nodes away from the root of the target segmental bronchus and vessels as completely as possible so that the bronchovascular bundle can be further identified in targeted segments.
2. Apply a rotatable stapler and adjust the angle by pulling the lung
  It is better to staple target segmental blood vessels and bronchi with a rotatable stapler and adjust the angle by pulling the lung to compensate for the limitation in access angle for the stapler. However, when segmental hilar structures are immediately below the field of vision, staplers are difficult to apply. In this case, a better choice is ligation and clipping directly.
3. Combined application of energy devices and staplers for the management of the intersegmental plane
  It is better to directly use electrocautery or ultrasonic scalpel to separate the inflation-deflation interface along the intersegmental veins up to the outer one-third of the pulmonary parenchyma. Once the distal stumps of the transected bronchus and vessels are dragged distantly from the hilum, the residual intersubsegmental parenchyma is easily tailored by staplers. This method can extend the residual lung and reduce air leakage.

In our study, 1 patient experienced a prolonged air leak and stayed in the hospital for 20 days after the operation. Air leakage is one of the major complications after segmentectomy [18]. The main causes of air leakage are (1) visceral pleural injury when dissecting the segmental vessels, bronchus and lymph nodes; (2) pulmonary parenchymal injury when energy devices are used for the management of the intersegmental plane; (3) air leakage at the nail holes of the stapler in patients with emphysema; and (4) bronchus injury. To reduce the occurrence of postoperative air leakage, it is necessary to separate the inflation-deflation interface along the intersegmental veins when separating the intersegmental plane. If bronchial injury is found, it must be sutured and repaired.

Preoperative 3D lung reconstruction can be used to clarify the anatomical structures, prejudge the variations and plan the surgical approach. Real-time 3D navigation can clearly identify the targeted bronchi and vessels and localize the nodules, which improves the accuracy of the surgical technique [19, 20].

This study has several limitations. First, this was a single-institute, retrospective review. Second, the sample size was small, so the statistical analysis was not robust. Finally, the definition of complex segmentectomy continues to be controversial. With the improvement in surgical experience and techniques, it is easy to resect the lung segments with their own anatomical structure except for segments 9 and 10. Therefore, we propose our own definition of complex segmentectomy, which makes several intersegmental planes or involves multiple target bronchi, arteries and veins. Our sample was limited and did not fully reflect the complexity of complex segmentectomy. Various resection methods for complex segmentectomy should be assessed further in future studies.

## Conclusion

In conclusion, our limited experience indicates that UP-VATS complex segmentectomy is a safe and effective procedure in expert hands that can spare more pulmonary parenchyma, ensure safe margins and provide the benefit of minimal invasiveness.

## Data Availability

The data and materials used in the current study are available from the corresponding author upon reasonable request.

## Abbreviations

VATS: Video-assisted thoracoscopic surgery
UP-VATS: Uniportal video-assisted thoracoscopic surgery
GGO: Ground glass opacity
CT: Computed tomography
3D: Three dimensional
RUL: Right upper lobe
LUL: Left upper lobe
RLL: Right lower lobe
LLL: Left lower lobe
AAH: Atypical adenomatous hyperplasia
AIS: Adenocarcinoma in situ
MIA: Minimal invasive adenocarcinoma
IAC: Invasive adenocarcinoma
